# Hydrogen Sulfide: A Therapeutic Candidate for Fibrotic Disease?

**DOI:** 10.1155/2015/458720

**Published:** 2015-05-11

**Authors:** Kai Song, Qian Li, Xiao-Ya Yin, Ying Lu, Chun-Feng Liu, Li-Fang Hu

**Affiliations:** ^1^Department of Nephrology, The Second Affiliated Hospital of Soochow University, Suzhou 215004, China; ^2^Institute of Neuroscience, Soochow University, Suzhou 215123, China; ^3^Department of Pharmacology, School of Pharmaceutical Science, Soochow University, Suzhou 215123, China

## Abstract

Fibrotic diseases including chronic kidney disease, liver cirrhosis, idiopathic pulmonary fibrosis, and chronic disease account for 45% mortality in the developed countries and pose a great threat to the global health. Many great targets and molecules have been reported to be involved in the initiation and/or progression of fibrosis, among which inflammation and oxidative stress are well-recognized modulation targets. Hydrogen sulfide (H_2_S) is the third gasotransmitter with potent properties in inhibiting inflammation and oxidative stress in various organs. Recent evidence suggests that plasma H_2_S level is decreased in various animal models of fibrotic diseases and supplement of exogenous H_2_S is able to ameliorate fibrosis in the kidney, lung, liver, and heart. This leads us to propose that modulation of H_2_S production may represent a promising therapeutic venue for the treatment of a variety of fibrotic diseases. Here, we summarize and discuss the current data on the role and underlying mechanisms of H_2_S in fibrosis diseases related to heart, liver, kidney, and other organs.

## 1. Introduction

Fibrotic disease refers to a group of clinical entities including chronic kidney disease, liver cirrhosis, idiopathic pulmonary fibrosis, and chronic heart failure, featured by chronic inflammatory diseases [1, 2]. As an important pathological feature, fibrosis is also present in many autoimmune diseases such as scleroderma, Crohn's disease, and systemic lupus erythematosus and affects the long-term survival of the graft as well as tumor metastasis patients [3]. In essence, fibrosis is a dysregulated wound healing process. It involves multiple cellular events such as the recruitment of inflammatory cells, the release of profibrotic cytokines, and the activation of collagen-producing cells including fibroblast, epithelial cells, and bone marrow stromal cells [4]. If highly progressive, fibrosis will eventually lead to the formation of permanent scars, irreversible organ dysfunction, and even death. In developed countries, fibrotic diseases account for nearly 45% morbidity and mortality [5]. Unfortunately, there are few effective therapies in most organ fibrosis, and the validated antifibrotic agents are even fewer. To date, the inhibitors of renin-angiotensin-aldosterone system (RAAS) are the primary medications for renal fibrosis and myocardial remodeling, but the application of these drugs is limited when serum creatinine rises above 3.5 mg/dL [6, 7]. Numerous novel therapeutic targets for fibrosis have been proposed, but monotarget therapy or simple combined treatment seems ineffective [8]. Since fibrosis is a disorder associated with multiple molecules and processes, small molecules interacting with several molecular targets of the fibrosis cascade would be promising for the treatment of fibrotic diseases.

Hydrogen sulfide (H_2_S) is the third gaseous transmitter secondary to nitric oxide (NO) and carbon monoxide [9]. For many decades, it was recognized as a poisonous gas because of its ability to inhibit cytochrome c oxidase in a similar manner to hydrogen cyanide. Recently, a lot of independent work demonstrates that H_2_S can be endogenously produced by cystathionine-*β*-synthase (CBS), cystathionine-*γ*-lyase (CSE), and 3-mercaptopyruvate sulphurtransferase (3-MST) in mammal organs and exerts important physiological and pathological effects. For instance, H_2_S is able to regulate vascular tone, inflammation, oxidative stress, cardiac contractility, nociception, insulin secretion, and resistance, and so forth. These have been intensively and extensively discussed and reviewed elsewhere [10]. Recently, it is interesting to find that plasma H_2_S level is decreased in many animal models of fibrotic diseases and the supplement of exogenous H_2_S partially reverses fibrosis in the fibrotic organs [11–13]. In this review, we summarize the recent evidence of the antifibrotic effect of H_2_S in various types of organs and discuss its underlying antifibrotic mechanisms, with the emphasis on its anti-inflammatory and antioxidative effects on fibrogenesis. Finally, we discuss the current obstacles for the application of H_2_S in the treatment of organ fibrosis.

## 2. Protective Effect of H_2_S against Fibrotic Diseases

### 2.1. H_2_S and Chronic Kidney Disease

Tubulointerstitial fibrosis is the final common pathway of chronic kidney disease (CKD) regardless of the initial injuries [14]. Pathologically, renal fibrosis is featured by the deposition of collagen I, collagen III, and collagen IV, the accumulation of myofibroblasts and extracellular matrix proteins, and the infiltration of inflammatory cells. Three H_2_S-producing enzymes, CBS, CSE, and 3-MST, have been found in various parts of the kidney and produce H_2_S in a synergistic way [15]. Recently, we and other researchers consistently demonstrated that CBS is the predominant H_2_S-generating enzyme located in proximal renal tubules, while CSE is sporadically expressed in glomeruli, renal arterioles, and interstitium [13, 16].

H_2_S plays physiological roles in maintaining normal function of kidney. For instance, H_2_S acts as an oxygen sensor monitoring the oxygen contents of the renal medulla and regulating the regional blood flow in renal cortex [17]. It also increases the glomerular filtration rate and exhibits diuretic property by excreting sodium and kalium ion into the urine [15].

Accumulating evidence suggests that H_2_S may inhibit fibrosis in CKD. The plasma H_2_S level has been found reduced in 5/6 nephrectomy rats and uremia patients [18, 19]. Moreover, in heterozygous *cbs* ^+/−^ mice with unilateral nephrectomy, a CKD model featured by proteinuria, the expressions of collagen and matrix metalloproteinase-2 and metalloproteinase-9 were markedly enhanced [20]. We recently found that, at a relatively lower dosage, H_2_S donor NaHS alleviated renal fibrosis in rats with unilateral urethral obstruction (UUO) [13]. Since UUO model is featured with progressive tubulointerstitial fibrosis without confounding causative factors such as proteinuria, hypertension, and uremia toxin, our study indicates a direct link between H_2_S and renal fibrosis. Moreover, CBS-derived H_2_S may be relevant in maintaining homeostasis in the kidney under normal conditions and may be suppressed under pathological conditions, because the amount of CBS protein level in proximal tubules was considerably and immediately reduced after UUO injury, while CSE expression in the renal interstitium was compensatorily enhanced and CSE inhibitor DL-propargylglycine (PAG) aggravated renal fibrosis [13]. This finding is consistent with a previous study, in which CBS reduction was earlier than the increase of CSE (6 h versus 24 h) after renal ischemic reperfusion [21]. Therefore, current data suggest that targeting CBS rather than CSE may be more promising in modulating endogenous H_2_S generation for the treatment of kidney fibrosis, particularly in the early period of renal fibrosis.

Another noteworthy effect of H_2_S on renal fibrosis is its inhibition on RAAS. The kidney contains all the components of RAAS. Angiotensin II is a potent profibrotic factor, which can stimulate collagen synthesis through the TGF-*β*1 [22] dependent and independent signaling pathway [23]. H_2_S can not only lower the serum angiotensin II level in renovascular hypertension animal model by downregulating the cellular cAMP production [24], but also counteract the hypertension, proteinuria, and renal damage induced by angiotensin II [25]. Recent data suggest that renin is also implicated in the progression of renal fibrosis and direct rennin inhibition with Aliskiren attenuating inflammation and fibrosis induced by UUO [26]. It has been found that H_2_S is able to inhibit the activity of angiotensin-converting enzyme (ACE) in human endothelial cells although the ACE mRNA expression is not altered [27]. Moreover, endogenous H_2_S can suppress the release of renin in As4.1 and renin-rich renal cells [28]. Taken together, all the data indicate that H_2_S may represent a novel venue for drug development to treat renal fibrosis, particularly when serum creatinine is beyond 3.5 mg/dL and the application of RAAS inhibitors is limited.

### 2.2. H_2_S and Hepatic Fibrosis

The liver is an important organ to maintain the plasma H_2_S homeostasis by regulating its production and elimination. Hepatic H_2_S production is mainly determined by CBS and CSE, both of which are rich in the liver. On the other hand, H_2_S is mainly eliminated in the liver via oxidation [29]. Similar to the kidney, CBS is more important in maintaining the normal liver function compared with CSE and 3-MST during physiological conditions [30].

The primary functions of H_2_S in the liver are associated with the regulations of lipid and glucose metabolism. H_2_S is critical for maintaining the normal lipid profile. The serum triglyceride and fatty acid levels were increased in* cbs* deficient mice [31], while high fat diet to* cbs* deficient mice enhanced serum total cholesterol and low density lipoprotein (LDL) cholesterol levels but decreased high density lipoprotein (HDL) cholesterol [32]. In addition, NaHS inhibited the insulin-stimulated absorption of glucose and decreased the glycogen content in HepG2 cells, indicating its beneficial effect on insulin resistance and diabetes [33].

Recent data also demonstrated that H_2_S may be implicated in hepatic fibrosis. Liver cirrhosis is associated with reduced serum H_2_S level. Tan et al. reported that NaHS (10 *μ*mol/kg, i.p.) significantly ameliorated liver fibrosis and portal hypertension induced by tetrachloride, while PAG (30 mg/kg, i.p.) resulted in converse effects [12]. Fan et al. also found that NaHS (500 *μ*M) inhibited hepatic stellate (HSC-T6) cell proliferation caused by ferric nitrilotriacetate [34]. However, the antifibrotic action of H_2_S on liver fibrosis may be of limited clinical relevance, because the primary causes of hepatic fibrosis are viral hepatitis and nonalcoholic fatty liver disease, not chemical intoxication. Moreover, inconsistent evidence stems from* cbs *genetic mutant mice. A study demonstrated that 3–8-week-old* cbs* deficient mice exhibited no signs of liver fibrosis [35], while another study revealed that the liver fibrosis was evident in 8–32-week* cbs* deficientmice [36]. Surely, it can be argued that the difference may be related to the mice age. As a result, more studies are needed to validate the antifibrotic effect of H_2_S in different animal models of hepatitis in the future.

### 2.3. H_2_S and Pulmonary Fibrosis

H_2_S can also be produced by CBS and CSE in the lung [37]. The expression pattern of the three H_2_S-producing enzymes varies among different species and cell types. In bovine pulmonary tissues, CSE is predominantly expressed in vascular smooth muscle cells (SMCs), while CBS is mainly located in endothelial cells [38]. In murid animals, CSE is found in blood vessel as well as airway SMCs in rat lung tissues, but CBS and CSE are colocalized in arterial and airway SMCs, endothelial cells, and SMCs [39]. Both CBS and CSE have been detected in human airway SMCs and lung fibroblast MRC5 cells [40].

H_2_S affects various respiratory system diseases. Serum H_2_S levels are significantly lower in chronic obstructive pulmonary disease patients than in control subjects [41]. H_2_S inhibits chronic inflammation, airway, and vascular remodeling and thus exhibits therapeutic effects on asthma and pulmonary hypertension [42, 43]. More importantly, the antifibrotic effect of H_2_S on pulmonary fibrosis has been recently reported. The serum H_2_S levels were decreased in the rats treated with bleomycin on day 7, compared with controls, while CSE mRNA expression was increased on days 7 and 28 [11]. Intraperitoneal injections with NaHS at 1.4 *μ*mol/kg and 7 *μ*mol/kg twice a day significantly reduced the contents of hydroxyproline and malondialdehyde (MDA) in the lung, although the significance was not obtained between these two dosages of NaHS [11]. Consistently, an* in vitro* study showed that 100 *μ*mol/L H_2_S inhibited the proliferation, migration, and transdifferentiation of human lung fibroblast stimulated by fetal bovine serum [44].

### 2.4. H_2_S and Cardiac Fibrosis

Adverse cardiac remolding is an important event that may eventually lead to chronic heart failure. Partial manifestation of cardiac remolding is the formation of interstitial fibrosis, featured by the activation of cardiac fibroblast associated with excessive formation of extracellular matrix within the myocardium. It is well known that the local RAAS is activated in chronic heart failure and plays an important role in cardiac remolding. Locally released angiotensin II is able to stimulate the proliferation of cardiac fibroblasts and increase the collagen production by activating the AT1 receptor [45]. Recent data suggests that aldosterone is also able to induce cardiac fibrosis by exhibiting proinflammatory effects and directly promoting the cardiac fibroblasts proliferation and collagen synthesis [46]. Although the production of cardiac renin is debatable, mast cells constitute a major source of renin after myocardial infarction, contributing to the formation of local angiotensin II [47].

H_2_S generation in the cardiovascular system is largely ascribed to CSE although CBS mRNA is detected in the tumoral tissue of the heart [48]. Similar to other organs, H_2_S is able to block cardiac fibrosis in various heart diseases. The first study on the association between cardiac fibrosis and H_2_S was presented by Shi et al., who demonstrated that exogenous administration of NaHS (10–90 *μ*mol/kg/day, i.p.) for three consecutive months markedly ameliorated the left ventricular remodeling and cardiac fibrosis in spontaneously hypertensive rat [49]. Similar results were obtained in streptozotocin-induced diabetic rats in which NaHS (14 *μ*mol/kg/day, i.p.) administration for 7 weeks attenuated the expressions of matrix metalloproteinase-2 (MMP-2), procollagen-1, and TGF-*β*1 in the left ventricle [50]. Moreover, administration with the same dose of NaHS (14 *μ*mol/kg/day, i.p.) for 14 days ameliorated collagen deposition in the left ventricle induced by abdominal aortic coarctation [51].

Recent evidence demonstrated that H_2_S exhibited antifibrotic effects in the heart by inhibiting the local RAAS. Exogenous H_2_S attenuated angiotensin II-induced hypertension, cardiac fibrosis, and oxidative stress in rats [52].* In vitro *study also confirmed the antiangiotensin II effect of H_2_S. For instance, NaHS (50–100 *μ*mol/L) inhibited the cardiac fibroblasts proliferation induced by 10% fetal bovine serum and angiotensin II [53]. Liu et al. reported that NaHS ameliorated isoproterenol-induced heart failure through the suppression of renin degranulation from cardiac mast cells [54]. As the renin level can be reduced by H_2_S, it is reasonable to hypothesize that H_2_S is able to decrease the aldosterone level in the heart. Future studies are needed to validate this hypothesis.

**Figure 1:**
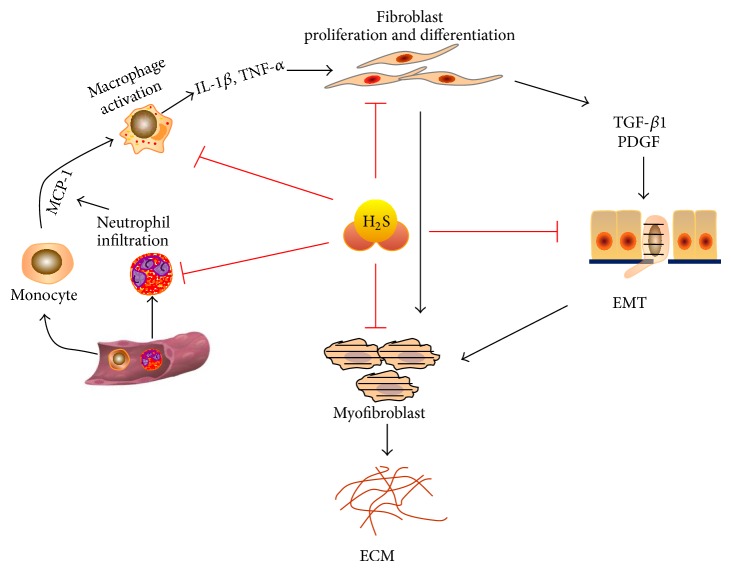
The effects of H_2_S on various cells associated with fibrosis. H_2_S inhibits neutrophil infiltration and macrophage activation during the inflammation response. H_2_S inhibits fibroblast proliferation and differentiation to myofibroblasts. In epithelial cells, H_2_S inhibits the epithelial-mesenchymal transition (EMT) process induced by various insults and cytokines. ECM: extracellular matrix.

The effects of H_2_S on various cells associated with fibrosis are summarized in Figure 1.

## 3. Cellular and Molecular Mechanisms of the Antifibrotic Effects of H_2_S

### 3.1. H_2_S Inhibits Myofibroblast Activation

Myofibroblast activation and proliferation are two key cellular events of fibrosis [4]. Once activated by injury or chronic inflammation, the fibroblast deposits extracellular matrix protein such as fibronectin within the cell and secrets fibrogenic cytokines such as TGF-*β*1, which in turn contributes to fibroblast differentiation. Although the origin of myofibroblasts is still debatable, a recent study using fate map technology revealed multiple origins of renal myofibroblasts, with 50% from resident fibroblasts, 35% from bone marrow, 10% arising from the endothelial-to-mesenchymal transition, and 5% from the epithelial-to-mesenchymal transition [55].

A multitude of* in vitro *studies demonstrated that H_2_S reduced the proliferation and differentiation of fibroblasts in various types of organs. In the kidney, NaHS at 100 *μ*mol/L suppressed the fetal bovine serum stimulated fibroblast proliferation by reducing DNA synthesis and the expressions of proliferating cell nuclear antigen and c-Myc within the cells. NaHS also blocked TGF-*β*1-induced fibroblasts differentiation through inhibiting the phosphorylation of Smad3 and mitogen-activated protein kinases [13]. Similar results were obtained in pancreatic stellate cells. NaHS (12.5–500 *μ*mol/L) decreased the cell number in a dose-dependent manner and halted the cell cycle progression at the G0/G1 check-point [56]. NaHS also inhibited the pancreatic stellate cells migration stimulated by 10% fetal bovine serum [56]. Lastly, in human alveolar epithelial cells (A549 cells), H_2_S (100 *μ*mol/L) reversed the TGF-*β*1-induced epithelial-mesenchymal transition by suppressing the expression of E-cadherin and increasing the vimentin levels within the cells [57].

### 3.2. H_2_S Exhibits Antifibrotic Effect by Inhibiting Inflammation

Inflammation plays a crucial role in the development and progression of fibrosis. The damaged epithelial cells and platelets may produce various chemotactic factors which further recruit neutrophils and macrophages to the damaged tissues [58]. Infiltration of these myeloid cells functions as a two-edged sword on fibrosis. On one hand, these cells are important for wound healing as they eliminate fibrin clots and cellular debris in the sites of injury. On the other hand, they secrete a variety of inflammatory mediators that may damage the surrounding tissues. If not properly eliminated, neutrophils and macrophages are capable of augmenting the inflammatory responses and eventually result in the formation of permanent scars.

It has been well recognized that macrophage serves as the most important immune cells associated with fibrosis. UUO animal model revealed a large quantity of macrophage infiltration in the renal interstitium in the early stage (3 days) after injury [59]. Macrophage ablation considerably reduced the production of various growth factors including TGF-*β*1 in various clinical settings [60]. Recent studies show that macrophage phenotype transition is implicated in modulating inflammation and fibrosis [61]. Classically activated macrophage (M1) exhibits proinflammatory and death-promoting effects [61]. Activated M1 cells also express peroxisome proliferator-activated receptor-*α* (PPAR-*α*) and PPAR-*γ* whose inhibitors have been proved to inhibit fibrosis progression [62]. By contrast, alternatively activated macrophage (M2) that is induced by IL-4 exhibits anti-inflammatory effects and promotes cell survival and proliferation [61]. Activated M2 cells also express enzyme arginase-1 (Arg1), through which M2 cells compete with T_help_ cells and myofibroblasts for L-arginine, and affect the production of L-proline and collagen [63].

A large body of evidence confirms the anti-inflammation property of H_2_S in various types of cells and organs, except at the super physiological range of concentrations. We verified that lower doses of NaHS (0.1–1 *μ*mol/kg/day, i.p.) reduced the infiltration of CD68 positive cells and decreased the expression of inflammatory cytokines including IL-1*β*, TNF-*α*, and monocyte chemoattractant protein-1 in the renal cortex at seven days after UUO injury. In contrast, CSE inhibitor PAG and higher dose of NaHS (10 *μ*mol/kg/day, i.p.) increased the number of macrophage in the renal interstitium [13]. In line with this, another study showed that NaHS (10 *μ*mol/kg/day, i.p.) markedly reduced the serum levels of TNF-*α*, IL-1*β*, IL-6, and soluble intercellular adhesion molecule (ICAM)-1 in carbon tetrachloride (CCl4) induced cirrhosis rats [12]. Apart from supplementation with NaHS, the modulations of endogenous H_2_S level with pharmacologic and genetic approaches* in vitro *also support the anti-inflammatory action of H_2_S in macrophage and other related cells. For example, we found that raw264.7 macrophage expressed CSE, and CSE upregulation inhibited the macrophage activation and TNF-*α* generation caused by oxidized low density lipoprotein through the suppression of nuclear transcription factor-*κ*B (NF-*κ*B) as well as the mitogen-activated protein kinase including the ERK1/2 and JNK [64]. In addition, Du et al. also reported that CBS overexpression or NaHS supplement could promote rotenone-treated microglia (central nervous system resident macrophage) transition from M1 toward M2 phenotype [65]. Thus, all the data suggest that H_2_S and its synthesis enzyme may be involved in regulating macrophage activation and its phenotype transition.

Apart from macrophage, neutrophils also participate in inflammation of fibrotic diseases. Evidence shows that neutrophils are present in most forms of glomerulonephritis (GN) such as acute poststreptococcal GN, immunoglobulin A nephropathy, and lupus nephritis [66]. Except a few forms of GN, most GN will eventually progress into renal fibrosis. Once recruited to the injury sites, neutrophils are activated via phagocytosis of immune complex or bacteria, generate reactive oxygen species (ROS), and degranulate multiple cytotoxic proteins including cationic serine proteases. Emerging evidence demonstrates that lower dose of NaHS is capable of reducing neutrophils infiltration and the damage in pancreas and decreasing the serum levels of P-selectin, E-selectin, ICAM-1, and VCAM-1 [67]. In support of this, plasma H_2_S levels are negatively correlated with the number of neutrophil and eosinophil in inflamed lung and joint disease [68]. Taken together, the evidence highly suggests that H_2_S may also exhibit its antifibrotic effect by suppressing neutrophils infiltration.

### 3.3. H_2_S Inhibits Fibrosis via Regulating Redox Homeostasis

ROS play crucial roles in the activation and differentiation of myofibroblasts [69]. NADPH oxidase (NOX) is a primary source of ROS by catalyzing the electron transmission from NADPH to oxygen. Such an effect differentiates NOX from other oxidases such as xanthine oxidase, which produces ROS as a by-product of their primary function [70]. There are seven types of NOX, among which NOX1, NOX2, and NOX4 are expressed in both rodent and human kidneys. Numerous studies show that NOX is associated with fibrotic diseases. NOX expression in fibroblasts can be induced by a variety of cytokines including TGF-*β*1, angiotensin II, and PDGF [71]. It has been reported that NOX expression was higher in lung fibroblasts isolated from idiopathic pulmonary patients than controls, and the colocalization of NOX and *α*-SMA was observed in samples from autoimmune hepatitis [72].* In vivo *studies also confirm the association of NOX and fibrosis. The NOX inhibitor or knockdown with siRNA ameliorated the fibrosis progression in renal, pulmonary, and liver fibrosis [15, 73, 74].

In addition to ROS, reactive nitrogen species (RNS) are also implicated in fibroblast-to-myofibroblast differentiation. In mammal cells, NO is generated by NO synthases (NOS) with L-arginine and oxygen as the substrate and NADPH as the cofactor. NO stimulates guanylyl cyclase (GC) and catalyzes guanosine triphosphate (GTP) to cyclic guanosine monophosphate (cGMP), which subsequently regulates the cGMP-dependent kinases (PKG) and enhances the intracellular calcium level [75]. NO signaling is critical for maintaining fibroblast phenotype. It has been found that TGF-*β*1 significantly decreased the NOS expression/activity and NO production in the dermal fibroblast while the NOS inhibitor *N*_*w*_-nitro-L-arginine methylester (L-NAME) enhanced the collagen contents within the cells. By contrast, the NO donor, sodium nitroprusside (SNP) inhibited the prostatic fibroblast differentiation into myofibroblast and L-arginine suppressed the TGF-*β*1-induced collagen production in dermal fibroblasts [76]. Consistently, the* in vivo* study also showed that NOS inhibition aggravated fibrosis in the kidney, heart, and penile [77–79].

Emerging data demonstrate that H_2_S acts as the scavenger of oxidant species. Notably, mammal cells have a high capacity of H_2_S oxidation in the mitochondria. The oxidation produces various products including persulfide, sulfite, thiosulfate (S_2_O_3_^2−^), and sulfate (SO_4_^2−^), most of which are excreted by the kidney in the form of sulfate [80].* In vitro *studies reveal that H_2_S donor (NaHS or Na_2_S) suppressed the protein oxidation caused by HOCl, ONOO^−^, and ^•^NO. Moreover, NaHS suppressed lipid oxidation and NOX activity. For example, NaHS reduced the MDA content in bleomycin-induced pulmonary fibrosis model, as well as the MDA formation in the lung tissue incubated with free radical-generating system* in vitro* [11]. In fact, H_2_S has been demonstrated to produce antioxidative effects in addition to acting as a direct ROS/RNS scavenger. For instance, Jung et al. reported that NaHS administration increased the expression/activity of catalase, copper-zinc superoxide dismutase, and manganese superoxide dismutase and also elevated the glutathione level in UUO animal model [81].

### 3.4. Energy-Sensing Molecules and H_2_S in Treating Fibrosis

Adipose tissue has been viewed as a passive fat storage depot; however, recent data suggest that adipose tissue is the largest endocrine organ of the body [82]. It secretes numerous cytokines that are involved in various physiological and pathological processes such as energy and cell metabolism, inflammation, oxidative stress, and fibrosis as well. Among the cytokines, adiponectin and its downstream signaling such as 5′-AMP-activated protein kinase (AMPK) have been highlighted in fibrotic diseases [82]. Classically, AMPK is activated when the cellular ATP is depleted under low caloric circumstances. Recently, mounting evidence shows that AMPK not only acts as an energy sensor, but also regulates redox signals. It is thus implicated in various pathological processes, such as neurodegeneration, cancer, and fibrosis. For example, acting through the AMPK pathway, adiponectin administration normalized the albuminuria and improved the podocyte function in adiponectin knockout mice [83]. Furthermore, AMPK activation with AICAR (5-aminoimidazole-4-carboxamide ribonucleotide) considerably reduced the mesangial matrix production and the urinary TGF-*β*1 levels [84]. AMPK activation also regulated the lipid accumulation and alleviated the kidney fibrosis induced by high fat diet [85, 86].

The metabolic inhibition property of H_2_S has been recently reported both* in vivo *and* in vitro*. It has been found that inhaled H_2_S induced a hibernation-like state in mice whose production of carbon dioxide and oxygen consumption were decreased approximately 90%. The core body temperature of the animal also reduced to the ambient temperature and their heart rates were relatively lower than the controls [86]. Exposure to various concentrations of NaHS reduced mitochondria membrane potential in renal proximal tubular cells (NRK52e) [87]. The mechanisms of the hypometabolism-inducing effect of H_2_S are not well classified. One hypothesis is that H_2_S inhibits cytochrome c oxidase and ATP generation and thus activates AMPK. We recently demonstrated that H_2_S activated AMPK in a calmodulin-dependent protein kinase kinase *β* (CaMKK*β*) manner, which is critical for the anti-inflammatory actions of H_2_S in the brain [88]. Similarly, a recent study reported that NaHS stimulated and restored AMPK phosphorylation which was reduced by high glucose in renal glomerular epithelial cells [89]. Other molecules may also be involved in the hypometabolism-inducing effect of H_2_S. For instance, sirtuin family members are important energy-related molecules as well as the downstream signal molecules of AMPK [90]. Sirtuins are mainly activated by calorie restriction and the deficiency of sirtuin perpetuates renal fibrosis and aging process [91, 92]. Recent data suggests that H_2_S increases the activity of sirtuin 1 induced by aging in human umbilical vein endothelial cell [93]. Therefore, H_2_S may produce antifibrotic effects by acting on the energy-sensing molecules.

**Figure 2:**
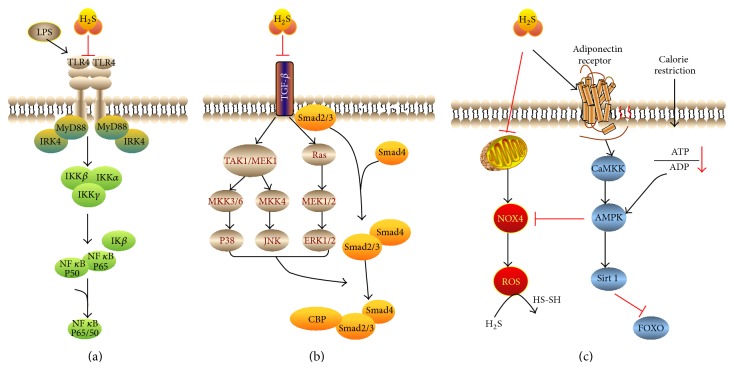
The schematic summarization for the signaling mechanisms of the antifibrotic effects of H_2_S. (a) H_2_S inhibits LPS-induced inflammation through the Toll-like receptor-NF *κ*B signal transduction. (b) H_2_S ablates the activation of TGF-*β*1 and MAPK kinases pathway. (c) H_2_S regulates redox production by inhibiting the expression/activity of NOX4 in addition to direct reaction with some reactive oxygen species (ROS). Lastly, H_2_S is able to modulate cellular metabolism and homeostasis by activating AMPK and sirtuin 1.

The antifibrotic mechanisms of hydrogen sulfide are presented in Figure 2.

## 4. Limitations and Perspectives

Although the antifibrotic property of H_2_S has been demonstrated in various animal models, current evidence mainly comes from the studies with the administration of exogenous H_2_S donor NaHS and Na_2_S in treating animal models of fibrotic diseases. The effect of endogenous H_2_S with genetically modified animals on organ fibrosis is still limited. The use of heterozygous *cbs* ^+/−^ mice with unilateral nephrectomy reveals the role of endogenous H_2_S in renal fibrosis, but its function in liver fibrosis is not confirmed [20, 36]. As the expression of CBS or CSE was reported to decrease in fibrotic diseases, the use of animals with overexpression of H_2_S-producing enzymes is needed in studying the effect of H_2_S on organ fibrosis. A transgenic mouse with CSE overexpression in the heart has been established and may be helpful in addressing the therapeutic effect of H_2_S on cardiac fibrosis in the future [94]. Recently, the intermediate-conductance Ca^2+^-activated K^+^ channel (K_Ca_3.1) has been proposed as the “switch” molecule of fibrotic disease because it regulates the proliferation, migration, and differentiation of renal and pulmonary fibrosis-producing cells [95–97]. Mouse with genetic mutation K_Ca_3.1 also confirms the antifibrotic effect of this ion channel in renal fibrosis [98]. As H_2_S is able to inhibit the big conductance of Ca^2+^-activated K^+^ channel (BK_Ca_) [99], it is interesting to further explore whether or not the antifibrotic effect of H_2_S correlates with the inhibition of K_Ca_3.1 ion channel.

Another limitation of the current studies is the difference in applied dose range of NaHS in combating fibrosis because the toxicity of H_2_S is always a concern. Lower doses of NaHS (1–10 *μ*mol/kg/day, i.p.) seem to be more effective in inhibiting renal, pulmonary, and liver fibrosis [11–13], but higher doses of NaHS (30 *μ*mol/kg/day, i.p.) are required for reversing the cardiac fibrosis in spontaneously hypertensive rats [49]. As the plasma H_2_S level in mammal tissues is still debatable, varying from 0.15 to 300 *μ*mol/L, due to the different measurements [100, 101], it is difficult to answer which dose of NaHS is mimicking the physiological relevance of H_2_S in animal tissues. Therefore, the development of fast, selective, and efficient detection method for sulfide monitoring is required for the booming research field of H_2_S biology.

Last but not least, the development of proper H_2_S-releasing agents is required to treat fibrotic diseases in a controlled way. GYY4137 and SG1002 are two orally administrated H_2_S-releasing compounds that have been proven to be beneficial in various diseases such as diabetes, hepatocellular carcinoma, and chronic heart failure [102–104]; however, their effects on fibrotic diseases have not been determined. More researches are therefore needed to explore the effectiveness of these and other similar agents on various fibrotic diseases.

## References

[1] Bataller R., Brenner D. A. (2005). Liver fibrosis. *The Journal of Clinical Investigation*.

[2] Wynn T. A. (2011). Integrating mechanisms of pulmonary fibrosis. *The Journal of Experimental Medicine*.

[3] Wynn T. A., Ramalingam T. R. (2012). Mechanisms of fibrosis: therapeutic translation for fibrotic disease. *Nature Medicine*.

[4] Liu Y. (2011). Cellular and molecular mechanisms of renal fibrosis. *Nature Reviews Nephrology*.

[5] Wynn T. A. (2008). Cellular and molecular mechanisms of fibrosis. *The Journal of Pathology*.

[6] Adamczak M., Gross M.-L., Krtil J. (2003). Reversal of glomerulosclerosis after high-dose enalapril treatment in subtotally nephrectomized rats. *Journal of the American Society of Nephrology*.

[7] Wolf G., Ritz E. (2005). Combination therapy with ACE inhibitors and angiotensin II receptor blockers to halt progression of chronic renal disease: pathophysiology and indications. *Kidney International*.

[8] Boor P., Šebeková K., Ostendorf T., Floege J. (2007). Treatment targets in renal fibrosis. *Nephrology Dialysis Transplantation*.

[9] Wang R. (2002). Two's company, three's a crowd: can H_2_S be the third endogenous gaseous transmitter?. *The FASEB Journal*.

[10] Wang R. (2012). Physiological implications of hydrogen sulfide: a whiff exploration that blossomed. *Physiological Reviews*.

[11] Fang L., Li H., Tang C., Geng B., Qi Y., Liu X. (2009). Hydrogen sulfide attenuates the pathogenesis of pulmonary fibrosis induced by bleomycin in rats. *Canadian Journal of Physiology and Pharmacology*.

[12] Tan G., Pan S., Li J. (2011). Hydrogen sulfide attenuates carbon tetrachloride-induced hepatotoxicity, liver cirrhosis and portal hypertension in rats. *PLoS ONE*.

[13] Song K., Wang F., Li Q. (2014). Hydrogen sulfide inhibits the renal fibrosis of obstructive nephropathy. *Kidney International*.

[14] Nangaku M. (2006). Chronic hypoxia and tubulointerstitial injury: a final common pathway to end-stage renal failure. *Journal of the American Society of Nephrology*.

[15] Xia M., Chen L., Muh R. W., Li P.-L., Li N. (2009). Production and actions of hydrogen sulfide, a novel gaseous bioactive substance, in the kidneys. *The Journal of Pharmacology and Experimental Therapeutics*.

[16] Bos E. M., Wang R., Snijder P. M. (2013). Cystathionine *γ*-lyase protects against renal ischemia/reperfusion by modulating oxidative stress. *Journal of the American Society of Nephrology*.

[17] Bełtowski J. (2010). Hypoxia in the renal medulla: implications for hydrogen sulfide signaling. *The Journal of Pharmacology and Experimental Therapeutics*.

[18] Aminzadeh M. A., Vaziri N. D. (2012). Downregulation of the renal and hepatic hydrogen sulfide (H 2S)-producing enzymes and capacity in chronic kidney disease. *Nephrology Dialysis Transplantation*.

[19] Perna A. F., Luciano M. G., Ingrosso D. (2009). Hydrogen sulphide-generating pathways in haemodialysis patients: a study on relevant metabolites and transcriptional regulation of genes encoding for key enzymes. *Nephrology Dialysis Transplantation*.

[20] Sen U., Basu P., Abe O. A. (2009). Hydrogen sulfide ameliorates hyperhomocysteinemia-associated chronic renal failure. *The American Journal of Physiology—Renal Physiology*.

[21] Wu N., Siow Y. L., Karmin O (2010). Ischemia/reperfusion reduces transcription factor Sp1-mediated cystathionine *β*-synthase expression in the kidney. *Journal of Biological Chemistry*.

[22] Wolf G., Zahner G., Schroeder R., Stahl R. A. K. (1996). Transforming growth factor beta mediates the angiotensin-II-induced stimulation of collagen type IV synthesis in cultured murine proximal tubular cells. *Nephrology Dialysis Transplantation*.

[23] Cuevas C. A., Gonzalez A. A., Inestrosa N. C., Vio C. P., Prieto M. C. (2014). Angiotensin II increases fibronectin and collagen I through the beta-catenin dependent signaling in mouse collecting duct cells. *The American Journal of Physiology—Renal Physiology*.

[24] Lu M., Liu Y.-H., Goh H. S. (2010). Hydrogen sulfide inhibits plasma renin activity. *Journal of the American Society of Nephrology*.

[25] Snijder P. M., Frenay A. R., Koning A. M. (2014). Sodium thiosulfate attenuates angiotensin II-induced hypertension, proteinuria and renal damage. *Nitric Oxide: Biology and Chemistry*.

[26] Choi D. E., Jeong J. Y., Lim B. J. (2011). Aliskiren ameliorates renal inflammation and fibrosis induced by unilateral ureteral obstruction in mice. *Journal of Urology*.

[27] Laggner H., Hermann M., Esterbauer H. (2007). The novel gaseous vasorelaxant hydrogen sulfide inhibits angiotensin-converting enzyme activity of endothelial cells. *Journal of Hypertension*.

[28] Lu M., Liu Y.-H., Ho C. Y., Tiong C. X., Bian J.-S. (2012). Hydrogen sulfide regulates cAMP homeostasis and renin degranulation in As4.1 and rat renin-rich kidney cells. *The American Journal of Physiology—Cell Physiology*.

[29] Norris E. J., Culberson C. R., Narasimhan S., Clemens M. G. (2011). The liver as a central regulator of hydrogen sulfide. *Shock*.

[30] Stipanuk M. H., Beck P. W. (1982). Characterization of the enzymic capacity for cysteine desulphhydration in liver and kidney of the rat. *The Biochemical Journal*.

[31] Namekata K., Enokido Y., Ishii I., Nagai Y., Harada T., Kimura H. (2004). Abnormal lipid metabolism in cystathionine *β*-synthase-deficient mice, an animal model for hyperhomocysteinemia. *The Journal of Biological Chemistry*.

[32] Mani S., Li H., Untereiner A. (2013). Decreased endogenous production of hydrogen sulfide accelerates atherosclerosis. *Circulation*.

[33] Zhang L., Yang G., Untereiner A., Ju Y., Wu L., Wang R. (2013). Hydrogen sulfide impairs glucose utilization and increases gluconeogenesis in hepatocytes. *Endocrinology*.

[34] Fan H.-N., Wang H.-J., Ren L. (2013). Decreased expression of p38 MAPK mediates protective effects of hydrogen sulfide on hepatic fibrosis. *European Review for Medical and Pharmacological Sciences*.

[35] Watanabe M., Osada J., Aratani Y. (1995). Mice deficient in cystathionine *β*-synthase: animal models for mild and severe homocyst(e)inemia. *Proceedings of the National Academy of Sciences of the United States of America*.

[36] Hamelet J., Demuth K., Paul J.-L., Delabar J.-M., Janel N. (2007). Hyperhomocysteinemia due to cystathionine beta synthase deficiency induces dysregulation of genes involved in hepatic lipid homeostasis in mice. *Journal of Hepatology*.

[37] Cismasiu V. B., Denes S. A., Reiländer H., Michel H., Szedlacsek S. E. (2004). The MAM (meprin/A5-protein/PTPmu) domain is a homophilic binding site promoting the lateral dimerization of receptor-like protein-tyrosine phosphatase mu. *The Journal of Biological Chemistry*.

[38] Olson K. R., Whitfield N. L., Bearden S. E. (2010). Hypoxic pulmonary vasodilation: a paradigm shift with a hydrogen sulfide mechanism. *American Journal of Physiology—Regulatory Integrative and Comparative Physiology*.

[39] Chen Y.-H., Wu R., Geng B. (2009). Endogenous hydrogen sulfide reduces airway inflammation and remodeling in a rat model of asthma. *Cytokine*.

[40] Perry M. M., Hui C. K., Whiteman M. (2011). Hydrogen sulfide inhibits proliferation and release of IL-8 from human airway smooth muscle cells. *The American Journal of Respiratory Cell and Molecular Biology*.

[41] Chen Y.-H., Yao W.-Z., Gao J.-Z., Geng B., Wang P.-P., Tang C.-S. (2009). Serum hydrogen sulfide as a novel marker predicting bacterial involvement in patients with community-acquired lower respiratory tract infections. *Respirology*.

[42] Bergeron C., Tulic M. K., Hamid Q. (2010). Airway remodelling in asthma: from benchside to clinical practice. *Canadian Respiratory Journal*.

[43] Chunyu Z., Junbao D., Dingfang B., Hui Y., Xiuying T., Chaoshu T. (2003). The regulatory effect of hydrogen sulfide on hypoxic pulmonary hypertension in rats. *Biochemical and Biophysical Research Communications*.

[44] Fang L.-P., Lin Q., Tang C.-S., Liu X.-M. (2009). Hydrogen sulfide suppresses migration, proliferation and myofibroblast transdifferentiation of human lung fibroblasts. *Pulmonary Pharmacology and Therapeutics*.

[45] Schorb W., Booz G. W., Dostal D. E., Conrad K. M., Chang K. C., Baker K. M. (1993). Angiotensin II is mitogenic in neonatal rat cardiac fibroblasts. *Circulation Research*.

[46] Lijnen P., Petrov V. (2000). Induction of cardiac fibrosis by aldosterone. *Journal of Molecular and Cellular Cardiology*.

[47] Mackins C. J., Kano S., Seyedi N. (2006). Cardiac mast cell-derived renin promotes local angiotensin formation, norepinephrine release, and arrhythmias in ischemia/reperfusion. *The Journal of Clinical Investigation*.

[48] Renga B. (2011). Hydrogen sulfide generation in mammals: the molecular biology of cystathionine-*β*-synthase (CBS) and cystathionine-*γ*-lyase (CSE). *Inflammation and Allergy—Drug Targets*.

[49] Shi Y.-X., Chen Y., Zhu Y.-Z. (2007). Chronic sodium hydrosulfide treatment decreases medial thickening of intramyocardial coronary arterioles, interstitial fibrosis, and ROS production in spontaneously hypertensive rats. *American Journal of Physiology: Heart and Circulatory Physiology*.

[50] El-Seweidy M. M., Sadik N. A. H., Shaker O. G. (2011). Role of sulfurous mineral water and sodium hydrosulfide as potent inhibitors of fibrosis in the heart of diabetic rats. *Archives of Biochemistry and Biophysics*.

[51] Huang J., Wang D., Zheng J., Huang X., Jin H. (2012). Hydrogen sulfide attenuates cardiac hypertrophy and fibrosis induced by abdominal aortic coarctation in rats. *Molecular Medicine Reports*.

[52] Snijder P. M., Frenay A. S., de Boer R. A. (2014). Exogenous administration of thiosulfate, a donor of hydrogen sulfide, attenuates Angiotensin II-induced hypertensive heart disease in rats. *British Journal of Pharmacology*.

[53] Liu J., Hao D. D., Zhu Y. C. (2011). Inhibitory effect of hydrogen sulfide on cardiac fibroblast proliferation. *Sheng Li Xue Bao*.

[54] Liu Y.-H., Lu M., Xie Z.-Z. (2014). Hydrogen sulfide prevents heart failure development via inhibition of renin release from mast cells in isoproterenol-treated rats. *Antioxidants and Redox Signaling*.

[55] Eddy A. A. (2013). The origin of scar-forming kidney myofibroblasts. *Nature Medicine*.

[56] Schwer C. I., Stoll P., Goebel U., Buerkle H., Hoetzel A., Schmidt R. (2012). Effects of hydrogen sulfide on rat pancreatic stellate cells. *Pancreas*.

[57] Fang L.-P., Lin Q., Tang C.-S., Liu X.-M. (2010). Hydrogen sulfide attenuates epithelial-mesenchymal transition of human alveolar epithelial cells. *Pharmacological Research*.

[58] Lee S. B., Kalluri R. (2010). Mechanistic connection between inflammation and fibrosis. *Kidney International. Supplement*.

[59] Chevalier R. L., Forbes M. S., Thornhill B. A. (2009). Ureteral obstruction as a model of renal interstitial fibrosis and obstructive nephropathy. *Kidney International*.

[60] Wynn T. A., Barron L. (2010). Macrophages: master regulators of inflammation and fibrosis. *Seminars in Liver Disease*.

[61] Gordon S., Taylor P. R. (2005). Monocyte and macrophage heterogeneity. *Nature Reviews Immunology*.

[62] Ricote M., Li A. C., Willson T. M., Kelly C. J., Glass C. K. (1998). The peroxisome proliferator-activated receptor-*γ* is a negative regulator of macrophage activation. *Nature*.

[63] Hesse M., Modolell M., la Flamme A. C. (2001). Differential regulation of nitric oxide synthase-2 and arginase-1 by type 1/type 2 cytokines in vivo: granulomatous pathology is shaped by the pattern of L-arginine metabolism. *Journal of Immunology*.

[64] Wang X. H., Wang F., You S. J. (2013). Dysregulation of cystathionine gamma-lyase (CSE)/hydrogen sulfide pathway contributes to ox-LDL-induced inflammation in macrophage. *Cellular Signalling*.

[65] Du C., Jin M., Hong Y. (2014). Downregulation of cystathionine *β*-synthase/hydrogen sulfide contributes to rotenone-induced microglia polarization toward M1 type. *Biochemical and Biophysical Research Communications*.

[66] Sorger K., Gessler U., Hübner F. K. (1982). Subtypes of acute postinfectious glomerulonephritis. Synopsis of clinical and pathological features. *Clinical Nephrology*.

[67] Sidhapuriwala J. N., Ng S. W., Bhatia M. (2009). Effects of hydrogen sulfide on inflammation in caerulein-induced acute pancreatitis. *Journal of Inflammation*.

[68] Whiteman M., Haigh R., Tarr J. M., Gooding K. M., Shore A. C., Winyard P. G. (2010). Detection of hydrogen sulfide in plasma and knee-joint synovial fluid from rheumatoid arthritis patients: relation to clinical and laboratory measures of inflammation. *Annals of the New York Academy of Sciences*.

[69] Sampson N., Berger P., Zenzmaier C. (2014). Redox signaling as a therapeutic target to inhibit myofibroblast activation in degenerative fibrotic disease. *BioMed Research International*.

[70] Altenhöfer S., Kleikers P. W. M., Radermacher K. A. (2012). The NOX toolbox: validating the role of NADPH oxidases in physiology and disease. *Cellular and Molecular Life Sciences*.

[71] Barnes J. L., Gorin Y. (2011). Myofibroblast differentiation during fibrosis: role of NAD(P)H oxidases. *Kidney International*.

[72] Jiang J. X., Chen X., Serizawa N. (2012). Liver fibrosis and hepatocyte apoptosis are attenuated by GKT137831, a novel NOX4/NOX1 inhibitor in vivo. *Free Radical Biology & Medicine*.

[73] Hecker L., Vittal R., Jones T. (2009). NADPH oxidase-4 mediates myofibroblast activation and fibrogenic responses to lung injury. *Nature Medicine*.

[74] Jarman E. R., Khambata V. S., Cope C. (2014). An Inhibitor of NADPH oxidase-4 attenuates established pulmonary fibrosis in a rodent disease model. *The American Journal of Respiratory Cell and Molecular Biology*.

[75] Murad F. (2006). Nitric oxide and cyclic GMP in cell signaling and drug development. *The New England Journal of Medicine*.

[76] Chu A. J., Prasad J. K. (1999). Up-regulation by human recombinant transforming growth factor *β*-1 of collagen production in cultured dermal fibroblasts is mediated by the inhibition of nitric oxide signaling. *Journal of the American College of Surgeons*.

[77] Sun D., Wang Y., Liu C., Zhou X., Li X., Xiao A. (2012). Effects of nitric oxide on renal interstitial fibrosis in rats with unilateral ureteral obstruction. *Life Sciences*.

[78] Kazakov A., Hall R., Jagoda P. (2013). Inhibition of endothelial nitric oxide synthase induces and enhances myocardial fibrosis. *Cardiovascular Research*.

[79] Ferrini M. G., Rivera S., Moon J., Vernet D., Rajfer J., Gonzalez-Cadavid N. F. (2010). The genetic inactivation of inducible nitric oxide synthase (iNOS) intensifies fibrosis and oxidative stress in the penile corpora cavernosa in type 1 diabetes. *The Journal of Sexual Medicine*.

[80] Kabil O., Motl N., Banerjee R. (2014). H2S and its role in redox signaling. *Biochimica et Biophysica Acta*.

[81] Jung K. J., Jang H. S., Kim J. I., Han S. J., Park J.-W., Park K. M. (2013). Involvement of hydrogen sulfide and homocysteine transsulfuration pathway in the progression of kidney fibrosis after ureteral obstruction. *Biochimica et Biophysica Acta—Molecular Basis of Disease*.

[82] Kershaw E. E., Flier J. S. (2004). Adipose tissue as an endocrine organ. *The Journal of Clinical Endocrinology and Metabolism*.

[83] Sharma K., RamachandraRao S., Qiu G. (2008). Adiponectin regulates albuminuria and podocyte function in mice. *The Journal of Clinical Investigation*.

[84] Declèves A.-E., Zolkipli Z., Satriano J. (2014). Regulation of lipid accumulation by AMK-Activated kinase in high fat diet-induced kidney injury. *Kidney International*.

[85] Declèves A.-E., Mathew A. V., Cunard R., Sharma K. (2011). AMPK mediates the initiation of kidney disease induced by a high-fat diet. *Journal of the American Society of Nephrology*.

[86] Blackstone E., Morrison M., Roth M. B. (2005). H_2_S induces a suspended animation-like state in mice. *Science*.

[87] Bos E. M., Leuvenink H. G. D., Snijder P. M. (2009). Hydrogen sulfide-induced hypometabolism prevents renal ischemia/reperfusion injury. *Journal of the American Society of Nephrology*.

[88] Zhou X., Cao Y., Ao G. (2014). CaMKK*β*-dependent activation of AMP-activated protein kinase is critical to suppressive effects of hydrogen sulfide on neuroinflammation. *Antioxidants & Redox Signaling*.

[89] Lee H. J., Mariappan M. M., Feliers D. (2012). Hydrogen sulfide inhibits high glucose-induced matrix protein synthesis by activating AMP-activated protein kinase in renal epithelial cells. *The Journal of Biological Chemistry*.

[90] Yang Z., Kahn B. B., Shi H., Xue B.-Z. (2010). Macrophage *α*1 AMP-activated protein kinase (*α*1AMPK) antagonizes fatty acid-induced inflammation through SIRT1. *The Journal of Biological Chemistry*.

[91] Cohen H. Y., Miller C., Bitterman K. J. (2004). Calorie restriction promotes mammalian cell survival by inducing the SIRT1 deacetylase. *Science*.

[92] Huang X. Z., Wen D., Zhang M. (2014). Sirt1 activation ameliorates renal fibrosis by inhibiting the TGF-beta/Smad3 pathway. *Journal of Cellular Biochemistry*.

[93] Suo R., Zhao Z.-Z., Tang Z.-H. (2013). Hydrogen sulfide prevents H_2_O_2_-induced senescence in human umbilical vein endothelial cells through SIRT1 activation. *Molecular Medicine Reports*.

[94] Elrod J. W., Calvert J. W., Morrison J. (2007). Hydrogen sulfide attenuates myocardial ischemia-reperfusion injury by preservation of mitochondrial function. *Proceedings of the National Academy of Sciences of the United States of America*.

[95] Roach K. M., Duffy S. M., Coward W., Feghali-Bostwick C., Wulff H., Bradding P. (2013). The K^+^ channel KCa3.1 as a novel target for idiopathic pulmonary fibrosis. *PLoS ONE*.

[96] Cruse G., Singh S. R., Duffy S. M. (2011). Functional KCa3.1 K^+^ channels are required for human fibrocyte migration. *The Journal of Allergy and Clinical Immunology*.

[97] Huang C., Shen S., Ma Q., Gill A., Pollock C. A., Chen X. M. (2014). KCa3.1 mediates activation of fibroblasts in diabetic renal interstitial fibrosis. *Nephrology Dialysis Transplantation*.

[98] Grgic I., Kiss E., Kaistha B. P. (2009). Renal fibrosis is attenuated by targeted disruption of KCa3.1 potassium channels. *Proceedings of the National Academy of Sciences of the United States of America*.

[99] Peers C., Bauer C. C., Boyle J. P., Scragg J. L., Dallas M. L. (2012). Modulation of ion channels by hydrogen sulfide. *Antioxidants and Redox Signaling*.

[100] Wintner E. A., Deckwerth T. L., Langston W. (2010). A monobromobimane-based assay to measure the pharmacokinetic profile of reactive sulphide species in blood. *British Journal of Pharmacology*.

[101] Zhong G., Chen F., Cheng Y., Tang C., Du J. (2003). The role of hydrogen sulfide generation in the pathogenesis of hypertension in rats induced by inhibition of nitric oxide synthase. *Journal of Hypertension*.

[102] Lu S., Gao Y., Huang X., Wang X. (2014). GYY4137, a hydrogen sulfide (H_2_S) donor, shows potent anti-hepatocellular carcinoma activity through blocking the STAT3 pathway. *International Journal of Oncology*.

[103] Kondo K., Bhushan S., King A. L. (2013). H_2_S protects against pressure overload-induced heart failure via upregulation of endothelial nitric oxide synthase. *Circulation*.

[104] Wei W.-B., Hu X., Zhuang X.-D., Liao L.-Z., Li W.-D. (2014). GYY4137, a novel hydrogen sulfide-releasing molecule, likely protects against high glucose-induced cytotoxicity by activation of the AMPK/mTOR signal pathway in H9c2 cells. *Molecular and Cellular Biochemistry*.

